# Suppression of breast cancer cell growth by Na^+^/H^+ ^exchanger regulatory factor 1 (*NHERF1*)

**DOI:** 10.1186/bcr1616

**Published:** 2006-11-01

**Authors:** Yong Pan, Lei Wang, Jia Le Dai

**Affiliations:** 1 Department of Molecular Pathology, The University of Texas M. D. Anderson Cancer Center, 7435 Fannin Street, Houston, TX 77054, USA

## Abstract

**Introduction:**

Na^+^/H^+ ^exchanger regulatory factor 1 *(NHERF1*, also known as *EBP50 *or *NHERF*) is a putative tumour suppressor gene in human breast cancer. Located at *17q25.1*, *NHERF1 *is frequently targeted during breast tumourigenesis. Loss of heterozygosity (LOH) at the *NHERF1 *locus is found in more than 50% of breast tumours. In addition, *NHERF1 *is mutated in a subset of primary breast tumours and breast cancer cell lines. LOH at the *NHERF1 *locus is strongly associated with aggressive features of breast tumours, implicating *NHERF1 *as a haploinsufficiency tumour suppressor gene. However, the putative NHERF1 tumour suppressor activity has not been functionally verified.

**Methods:**

To confirm the NHERF1 tumour suppressor activity suggested by our genetic analyses, we used retrovirus-transduced short hairpin RNA (shRNA) to knock down NHERF1 expression in breast cancer cell lines MCF7 and T47D. These cells were then assessed for cell growth *in vitro *and *in vivo*. The control and NHERF1 knockdown cells were also serum-starved and re-fed to compare their cell cycle progression as measured by fluorescence-activated cell sorting analyses.

**Results:**

We found that downregulation of the endogenous NHERF1 in T47D or MCF7 cells resulted in enhanced cell proliferation in both anchorage-dependent and -independent conditions compared with that of the vector control cells. NHERF1 knockdown T47D cells implanted at mammary fat pads of athymic mice formed larger tumours than did control cells. We found that serum-starved NHERF1 knockdown cells had a faster G_1_-to-S transition after serum re-stimulation than the control cells. Immunoblotting showed that the accelerated cell cycle progression in NHERF1 knockdown cells was accompanied by increased expression of cyclin E and elevated Rb phosphorylation level.

**Conclusion:**

Our findings suggested that the normal NHERF1 function in mammary epithelial cells involves blockage of cell cycle progression. Our study affirmed the tumour suppressor activity of NHERF1 in breast which may be related to its regulatory effect on cell cycle. It warrants future investigation of this novel tumour suppressor pathway in human breast cancer which may turn up therapeutic opportunities.

## Introduction

Na^+^/H^+ ^exchanger regulatory factor 1 (*NHERF1*, also known as *EBP-50 *or *NHERF*) is a candidate tumour suppressor gene in human breast cancer [1]. We reported loss of heterozygosity (LOH) at the *NHERF1 *gene locus (*17q25.1*) in more than 50% of human breast tumours. Such loss is infrequent, however, in other tumour types, suggesting that *NHERF1 *is specifically targeted during mammary tumourigenesis. In a panel of breast tumours pre-screened for LOH, three intragenic mutations of *NHERF1 *were found (approximately 3%) [1]. LOH at the *NHERF1 *locus is positively correlated with aggressive features of breast tumours, including tumour size, grade, and stage. The association indicates a critical role for *NHERF1 *in mammary carcinogenesis, in which its putative suppressor activity is haploinsufficient. The haploinsufficiency of the *NHERF1 *gene may explain its relatively low frequency of intragenic mutations.

The *NHERF1 *gene encodes an intracellular molecule that was initially found to be a cofactor necessary for cAMP-mediated inhibition of renal apical Na^+^/H^+ ^exchanger isoform 3 (NHE3) [2]. Human NHERF1 is a 358-amino acid protein that shares high homologue at the modular structures with NHERF2 (also known as E3KARP or TKA1) [3]. Both contain two tandem PDZ (PSD-95/Dlg/ZO1) domains (PDZ-I and PDZ-II) at the amino-terminus and an ezrin-radixin-moesin (ERM)-interacting domain at the carboxyl-terminus. NHERF1 and NHERF2 are highly expressed in polarised epithelial cells and are differentially expressed in mammalian tissues [2]. NHERF1, the one more extensively studied, acts as an important regulator and integrator of multiple signaling pathways by virtue of its ability to bind to a variety of proteins through its PDZ domains and an ERM-interacting domain. Via its PDZ domains, NHERF1 specifically recognises carboxyl-terminal motif (S/T)XL, which is present in a number of transmembrane proteins other than NHE3, including cystic fibrosis transmembrane conductance regulator [4-6], β_2_-adrenergic receptor [7,8], platelet-derived growth factor receptor (PDGFR) [9,10], and sodium bicarbonate co-transporter [11]. NHERF1 has also been shown to interact with a variety of intracellular proteins, including phospholipase C-β isoforms [12,13], GRK6A (G protein-coupled receptor kinase 6A) [14], spleen tyrosine kinase (SYK) [1], YAP65 (Yes-associated protein 65-kDa) [15], and β-catenin [16]. The proteins recognised by PDZ-I do not, however, bind to PDZ-II, and *vice versa*, indicating that the two PDZ domains have distinct binding motifs [4,14].

NHERF1 binds, via its ERM-binding domain, to ERM proteins, a family of actin cytoskeletal adaptor proteins [17,18]. One ERM family member is merlin, the product of neurofibromatosis-2 (*NF2*), a tumour suppressor gene implicated in predisposition to meningiomas and schwanomas [19,20]. The amino-terminus of the ERM family proteins (ERM domain) interacts with the ERM-binding domain of NHERF1 [17,18]. The interaction may be important for NHERF1 functions by connecting membrane transporters and actin cytoskeleton [21,22]. Like other ERM members, merlin interacts with NHERF1 through its amino-terminus ERM domain. Notably, more than 80% of *NF2 *mutations are located in this ERM domain [23], and the mutant merlin proteins display significantly lower binding affinity to NHERF1, suggesting that NHERF1 is related to merlin's suppressor activity.

Among the multiple biologic pathways in which NHERF1 is involved, the signaling event that is most relevant to NHERF1 pathobiology in mammary gland is not known, nor is it certain that NHERF1 elicits tumour suppressor activity in breast. Human *NHERF1 *was earlier shown to be an oestrogen-inducible gene [24,25]. Based on a critical role of oestrogen in mammary development and the early-stage progression of breast cancer, NHERF1 was initially postulated as a mitogenic factor [22], which is not supported by our genetic evidence [1]. To clarify these contrasting views, we sought to determine whether the proliferation of breast cancer cells is affected by knockdown of NHERF1 expression.

## Materials and methods

### Cell culture

Human breast tumour cell lines BT20, BT474, BT483, BT549, CAMA1, DU4475, HCC1428, HCC1954, MB157, MCF7, MDA-MB-134, MDA-MB-231, MDA-MB-330, MDA-MB-361, MDA-MB-415, MDA-MB-435S, MDA-MB-453, MDA-MB-468, SKBr3, T47D, and ZR75-1 were purchased from American Type Culture Collection (Manassas, VA, USA). The SUM149-PT line was a gift from Dr. Stephan Ethier (University of Michigan, Ann Arbor, MI, USA). All cell lines were cultured in recommended media supplemented with 10% foetal bovine serum (FBS) (Invitrogen, Carlsbad, CA, USA).

### Knockdown of NHERF1 expression

A vector-based short hairpin RNA (shRNA) method was used to generate MCF7 and T47D cells with inhibited NHERF1 expression. A two-step ligation method [26] was used to insert the interfering sequences into pBS/U6 (a gift from Dr. Yang Shi, Harvard Medical School, Boston, MA, USA). Two NHERF1 mRNA sequences corresponding to cDNA positions 786 and 910 were targeted. Oligonucleotide sequences for NHERF-786 were 5'-GGAGATACAGAAGGAGAACAGA-3' (oligo 1, forward), 5'-AGCTTCTGTTCTCCTTCTGTATCTCC-3' (oligo 1, reverse), 5'-AGCTTCTGTTCTCCTTCTGTATCTCCCTTTTTG-3' (oligo 2, forward), and 5'-AATTCAAAAAGGGAGATACAGAAGGAGAACAGA-3' (oligo 2, reverse). Oligonucleotide sequences for NHERF-910 were 5'-GGAAACTGACGAGTTCTTCAA-3' (oligo 1, forward), 5'-AGCTTTGAAGAACTCGTCAGTTTCC-3' (oligo 1, reverse), 5'-AGCTTTGAAGAACTCGTCAGTTTCCCTTTTTG-3' (oligo 2, forward), and 5'-AATTCAAAAAGGGAAACTGACGAGTTCTTCAA-3' (oligo 2, reverse). Interference sequences were verified by automated DNA sequencing. The hairpin loop sequences were then released by digesting with *Bam*HI and *Eco*RI and subcloned into a retroviral vector pBabe-U6 (a gift from Dr. Jinsong Liu, M. D. Anderson Cancer Center, Houston, TX, USA) [27], yielding pBabe-U6/NHERF-786 and pBabe-U6/NHERF-910. Retroviruses were produced by transfecting packaging cells (amphotropic Phoenix) with pBabe-U6/NHERF-786, pBabe-U6/NHERF-910, or parental pBabe-U6, using Fugene 6 (Roche Applied Science, Indianapolis, IN, USA). The medium was collected 2 days after transfection. After centrifugation, the supernatant was then passed through a 0.45-μm filter. The retrovirus stock was stored at -80°C until use. Cultured MCF7 and T47D cells were infected with a virus cocktail (1 ml of retroviral stock, 2 ml of medium, and 4 μg of polybrene). The next day, the virus was removed and replaced with fresh medium that contained 0.5 μg/ml puromycin. Surviving cells were assessed for NHERF1 expression by immunoblotting.

### Cell growth assay

Thymidine incorporation assay was used to measure the DNA synthesis rate as described previously [26]. MCF7 and T47D cells cultured in 24-well plates were pulsed with 1 mCi [^3^H]-thymidine (3,000 Ci/mmol; PerkinElmer Life and Analytical Sciences, Inc., Shelton, CT, USA). After 5-hour labeling, non-incorporated tritium was removed by trichloroacetic acid washes. Acid-insoluble tritium was assessed by scintillation counting (microBeta Trilux 1450; Wallac, now PerkinElmer Life and Analytical Sciences, Inc.). The relative cell proliferation rate was obtained by dividing the counts from cells in which NHERF1 was downregulated by the ones from control cells. Experiments were repeated three times.

3-(4,5-Dimethylthiazol-2-yl)-2,5-diphenyltetrazolium bromide (MTT) assays were conducted to measure the relative number of viable cells. Cells were seeded in 96-well cluster dishes at 2,500 cells per well with 100 μl of complete medium. At indicated time points, medium was replaced with 100 μl of fresh medium supplemented with 20 μl of 5 mg/ml MTT (Sigma-Aldrich, St. Louis, MO, USA). The incubation lasted for 2 hours before the medium was removed and cells dissolved in 100 μl of lysis buffer. Absorbance was measured using a multiSkan plate reader (Thermo Scientific, Waltham, MA, USA) at a wavelength of 570 nm. Experiments were repeated at least three times.

### Anchorage-independent growth

Cells (1 × 10^4^) were suspended in 1 ml of 1× culture medium that contained 0.35% agarose. The suspension was added on top of 4 ml of solidified 0.7% agarose. After the cells were set in agarose, 1 ml of fresh medium was added to cover the agarose. Assays were performed in triplicate. Plated cells were incubated for 20 days at 37°C before formed colonies larger than 50 μm in diameter were counted. Experiments were repeated three times.

### Assessment of cell cycle distribution

Cultured cells (approximately 2 × 10^6^) were trypsinised and washed twice with 1× phosphate-buffered saline (PBS). Cells were then fixed by being added drop-wise to 5 ml of ice-cold 80% ethanol while vortexing. After fixing for at least 1 hour at room temperature, the cells were stored at -20°C. Before being stained, the cells were washed with 1× PBS and incubated at 37°C for 30 minutes with propidium iodide (50 μg/ml; Sigma-Aldrich) in the presence of 10 μl of RNase A (10 mg/ml; Sigma-Aldrich). Cell cycle analysis was performed with an FACS station equipped with CellQuest (Becton Dickinson, Franklin Lakes, NJ, USA). At each cell cycle phase, the population was determined by computer model fitting (Verity Software House, Topsham, ME, USA).

Serum starvation was used to synchronise MCF7 at the G_0_/G_1 _phase. MCF7 cells were seeded at 8 × 10^5 ^per 60-mm dish. After being cultured in complete medium overnight, cells were incubated with serum-free medium for 1 day. The cells were then re-fed with medium supplemented with 10% FBS for various time periods before being harvested for fluorescence-activated cell sorting (FACS) analyses.

### Experimental tumourigenicity assay

Four- to five-week-old female athymic nude mice (Harlan, Indianapolis, IN, USA) were used for experimental tumourigenicity assays. To facilitate the establishment of xenografts of oestrogen-dependent cells, each mouse was inoculated subcutaneously with an oestrogen pellet (0.7 mg 17β-estradiol per pellet; 60-day slow-release; Innovative Research of America, Sarasota, FL, USA). Two days after pellet implantation, equivalent amounts of T47D cells (1.5 × 10^6^; Babe control or NHERF-910) resuspended in 100 μl of mixture (1:1 with un-supplemented media) of Matrigel (BD Biosciences, San Jose, CA, USA) were injected into each side of second-pair breast mammary fat pads (3 × 10^6 ^cells in total). Six weeks after injection, mice were euthanised by carbon dioxide, and the established tumours on both sides of mammary glands were dissected, pooled, and weighed. All procedures were performed according to the recommendations of the Institutional Animal Care and Use Committee.

### Immunoblotting

Immunoblottings were carried out essentially as described previously [28]. Antibodies used were NHERF1 (EXBIO Praha, Bestec, Czech Republic), Rb and p27 (BD Biosciences), cdk2 (Calbiochem, San Diego, CA, USA), cdk4 and cyclin D1 (Cell Signaling Technology, Inc., Danvers, MA, USA), cyclin E and β-actin (Santa Cruz Biotechnology, Inc., Santa Cruz, CA, USA), and α-tubulin (Sigma-Aldrich).

## Results

### NHERF1 expression in breast cancer cells

**Figure 1:**
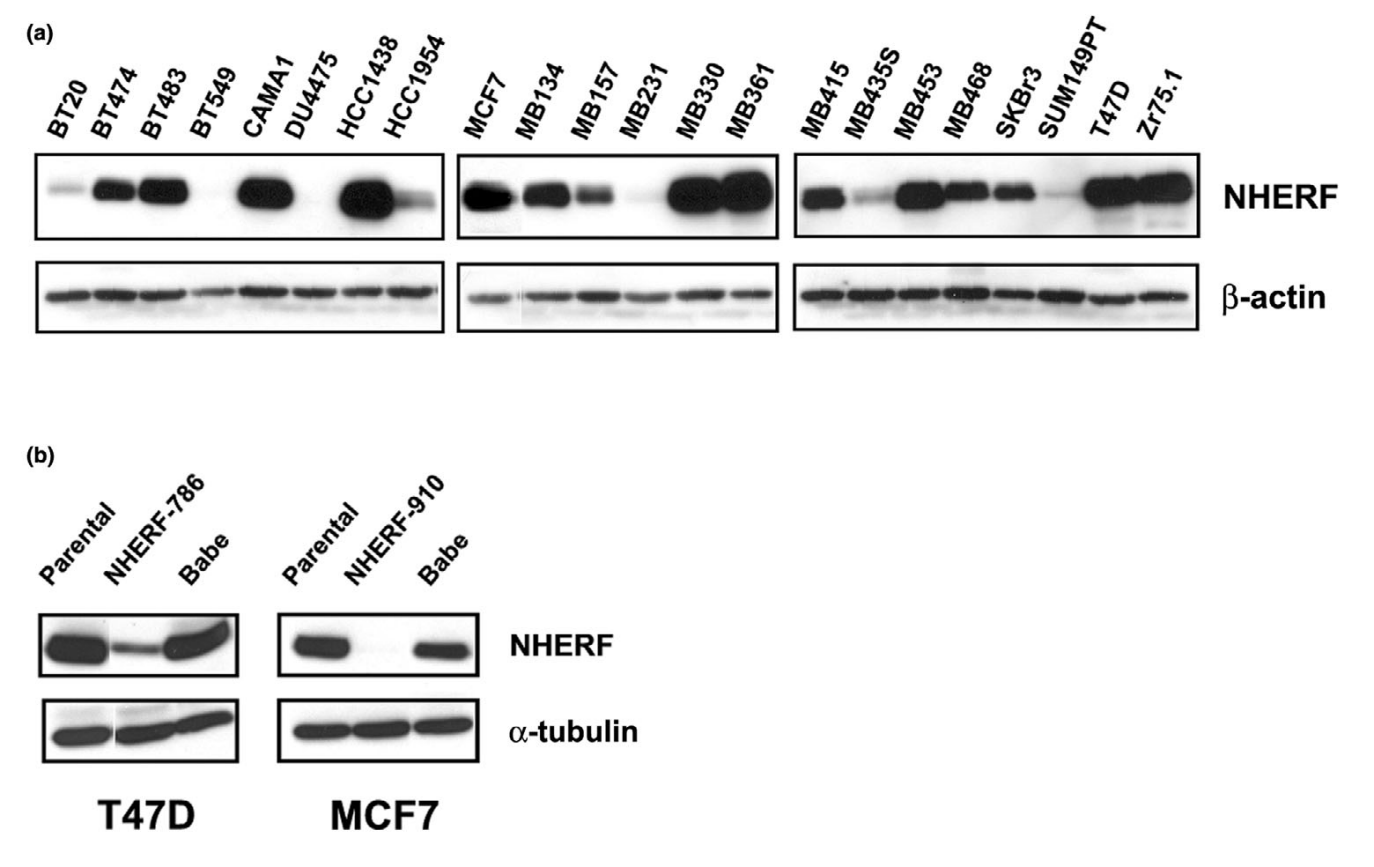
Knockdown of Na^+^/H^+ ^exchanger regulatory factor 1 (NHERF1) expression in breast cancer cells. **(a) **Breast cancer cell lines, as indicated, were analysed for NHERF1 expression by immunoblotting. Blots were stripped and re-probed with β-actin for loading control. **(b) **Short hairpin RNA (shRNA) sequences targeting two different positions of human NHERF1 mRNA, NHERF-786 and NHERF-910, were retrovirally transduced in T47D and MCF7 cells, as shown. Cells were also infected with empty virus (Babe) to be used as a control. Cells that expressed NHERF1 shRNA sequences were selected by 0.5 μg/ml of puromycin. NHERF1 expression in MCF7 and T47D stable cells and their parental lines was examined by immunoblotting. α-Tubulin blotting was used for normalisation.

We used immunoblotting to analyse the expression of NHERF1 in 22 breast cancer cell lines (Figure 1a), among which the origin of MDA-MB-435S was under debate [29-31]. MDA-MB-231 and SUM149-PT, two lines harbouring both NHERF1 mutation and LOH [1], had low NHERF1 protein levels. High expression of NHERF1 was found in some oestrogen receptor (ER)-α-positive cells, such as T47D, Zr75.1, and MCF7, consistent with an earlier report that correlated NHERF1 expression with ER-α positivity [32]. However, NHERF1 expression does not necessarily follow the ER-α status. For example, BT20 cells expressing constitutively active ER-α had low NHERF1 expression, whereas NHERF1 was maintained at a high level in ER-α-negative MDA-MB-453, MDA-MB-468, and SKBr3 cells, suggesting that mechanisms other than oestrogen induction must exert an effect on NHERF1 expression.

### Growth property changes in response to NHERF1 knockdown

One way to determine whether NHERF1 functions as a tumour suppressor gene in human breast cancer is to assess the resultant phenotypic responses by knocking down endogenous NHERF1 expression in NHERF1-high expressors. We used a retrovirus-based system to deliver shRNA to knock down NHERF1 expression in T47D cells. Introduction of NHERF1 shRNA-786 markedly lowered the NHERF1 level (Figure 1b, left panel). By contrast, NHERF1 expression was unaffected by empty retrovirus (Babe). ShRNA can sometimes create non-specific outcomes that are not related to the targeted gene of interest [33]. To ascertain the specificity of phenotypic changes resulting from NHERF1 loss, we also prepared an NHERF1 knockdown line from MCF7 cells by introducing a different targeting sequence, NHERF1 shRNA-910. Introducing shRNA-910 led to virtual elimination of NHERF1 expression in MCF7 cells, in comparison with that of parental cells and Babe control (Figure 1b, right panel).

**Figure 2:**
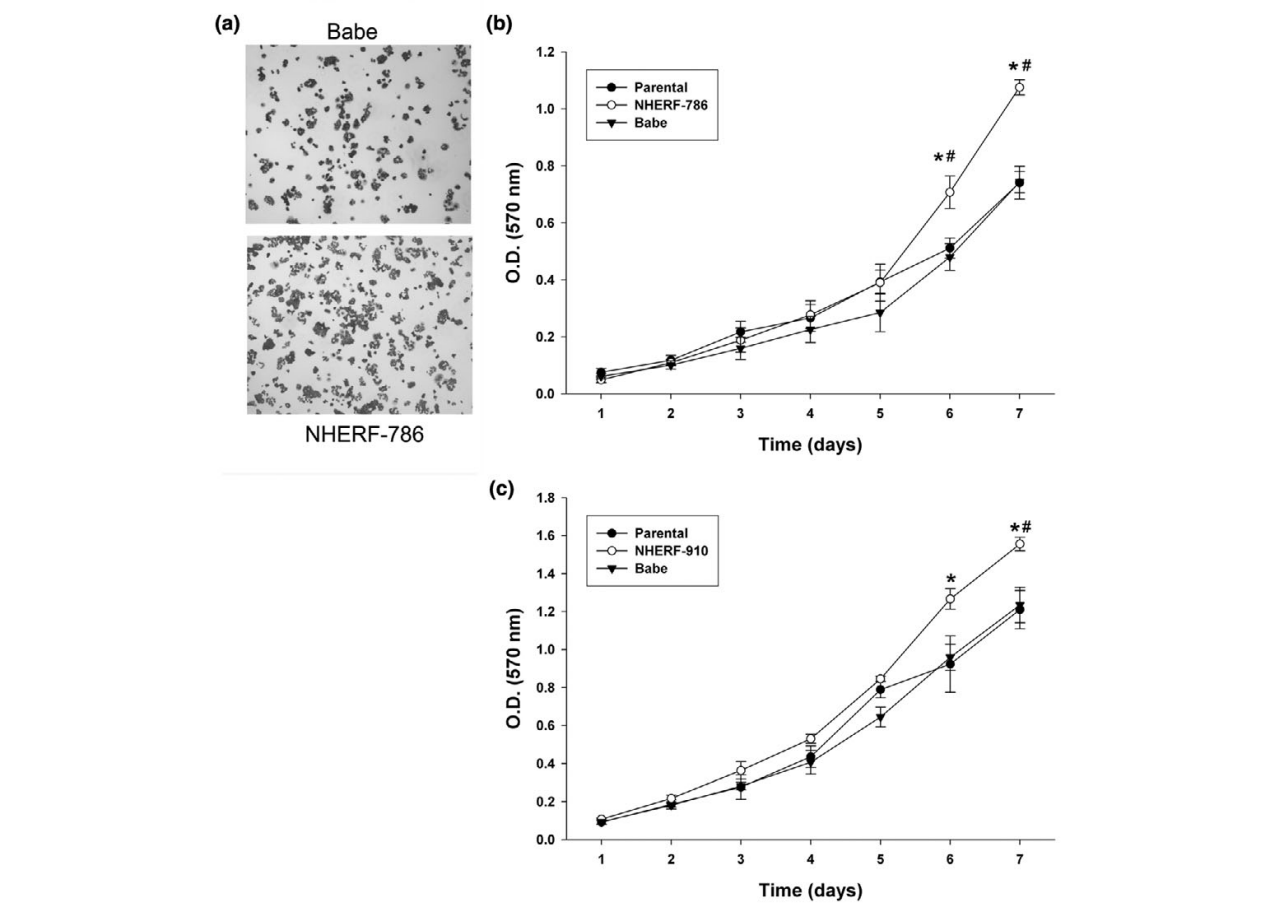
Growth of T47D and MCF7 cells with Na^+^/H^+ ^exchanger regulatory factor 1 (NHERF1) knockdown. **(a) **Representative phase-contrast view of monolayer culture of T47D/NHERF-786 cells in comparison with T47D/Babe control, prior to MTT assay. **(b) **Growth-rate comparison of T47D/NHERF-786 and T47D/Babe cells by MTT assay. Cells cultured in 96-well plates were allowed to grow for 1 to 7 days before MTT reagents were added. The metabolic products of MTT deposited in cells were measured by optical density (O.D.) at 570 nm. **(c) **MCF7/NHERF-910 and MCF7/Babe cells were measured by MTT assay as described in **(b)**. The growth curve is representative of at least three independent experiments. *P < 0.05 versus Babe cells. #*P *< 0.05 versus parental cells. MTT, 3-(4,5-dimethylthiazol-2-yl)-2,5-diphenyltetrazolium bromide.

One frequent response of a given tumour suppressor gene is to inhibit cell proliferation. To determine whether NHERF1 affects cell proliferation, we sought to compare the growth curve of T47D/NHERF-786 and MCF7/NHERF-910 cells with that of their corresponding Babe controls, as determined by MTT assay in 96-well plates. As shown in Figure 2a and 2b, although infection of Babe retrovirus did not affect cell proliferation (parental T47D versus T47D/Babe cells), T47D/NHERF-786 cells grew consistently faster than T47D/Babe control cells, most obviously at days 6 and 7 after plating (by approximately 35%). Similarly, the growth rate of MCF7/NHERF-910 was found to be higher (by 30% to 40%) than that of MCF7/Babe or parental MCF7 cells (Figure 2c). These results suggested an inhibitory effect of NHERF1 on cell proliferation.

**Figure 3:**
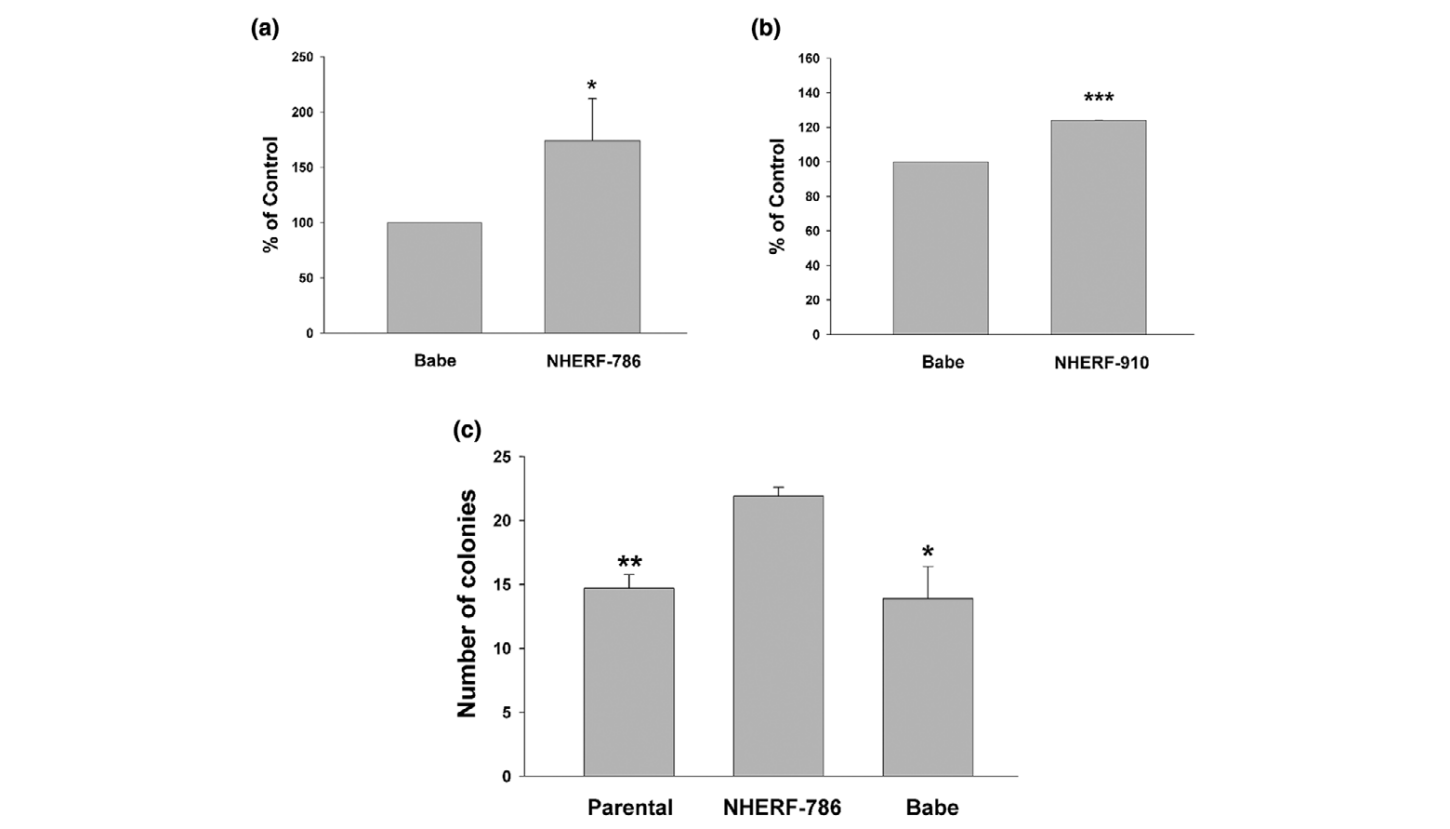
Lowered Na^+^/H^+ ^exchanger regulatory factor 1 (NHERF1) expression leads to increased proliferation in an anchorage-dependent and -independent manner. **(a,b) **NHERF1 knockdown T47D **(a) **and MCF7 **(b) **cells were pulsed with [^3^H]-thymidine to assay for DNA incorporation. Data are presented as means ± standard error, arbitrarily setting Babe at 100%. The average of three independent experiments was plotted. *P < 0.05, ***P < 0.001. **(c) **Soft-agar assay. Parental T47D, Babe control, and NHERF-786 knockdown cells (1 × 10^4^) were suspended in 1 ml of 1× culture medium that contained 0.35% agarose. The suspension was added on top of 4 ml of solidified 0.7% agarose. Plated cells were incubated for 20 days at 37°C. Formed colonies larger than 50 μm in diameter were counted. Assays were performed in triplicate. The average of three independent experiments is presented. *P < 0.05, **P < 0.01, in comparison with T47D/NHERF-786.

The DNA synthesis of these cells was also compared using [^3^H]-thymidine incorporation assays. Knockdown of NHERF1 in T47D cells resulted in a significantly higher DNA synthesis rate than that of the Babe control cells (*P *= 0.022; Figure 3a). MCF7 cells with NHERF1 knockdown consistently showed accelerated proliferation (*P *= 4 × 10^-6^; Figure 3b). The regulatory effect of NHERF1 on cell proliferation was also reflected in anchorage-independent growth. T47D/NHERF-786 cells showed a more efficient colony outgrowth in soft agar than did either parental T47D (*P *= 0.0043) or T47D/Babe (*P *= 0.020) cells (Figure 3c), indicating that NHERF1 exhibits growth suppression activity in breast cancer cells.

### Tumour growth properties *in vivo*

**Figure 4:**
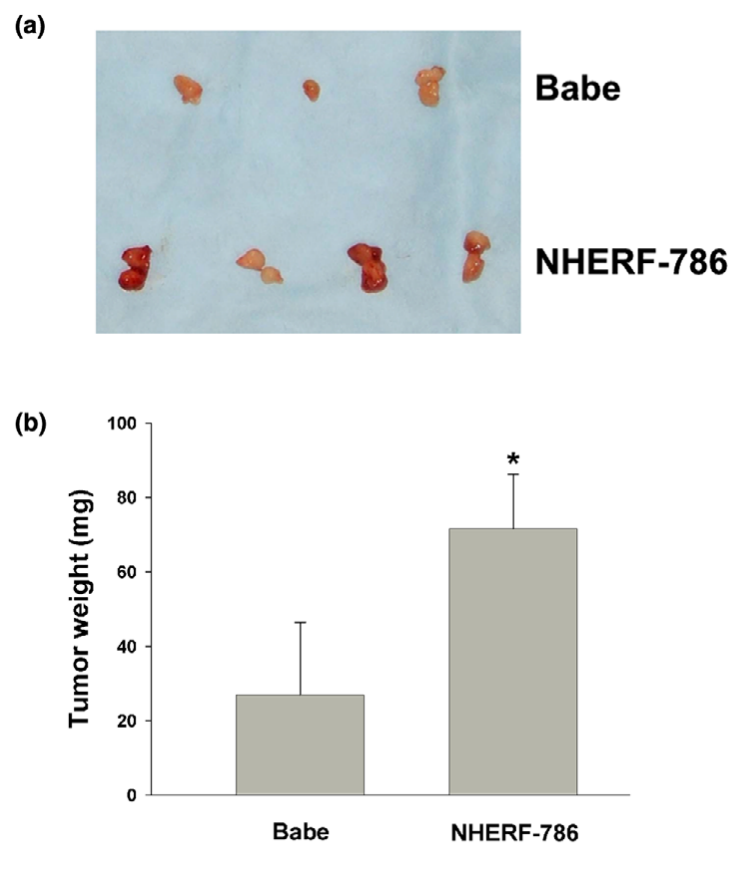
Tumour growth after injection into nude mice. Athymic nude mice were pre-implanted subcutaneously with oestrogen pellets to support tumour establishment. Three tenths of a million T47D/Babe (*n *= 10) or T47D/NHERF-786 (*n *= 8) cells were injected into both sides of the mammary fat pads of each nude mouse (1.5 × 10^6 ^on each side). Forty-two days after injection, tumours were dissected **(a) **and weighed for growth comparison **(b)**. Data are presented as means ± standard error. *P < 0.05.

The growth-promotion response as result of NHERF1 knockdown was also examined in a mouse xenograft model. We compared the *in vivo* growth of T47D cells infected with NHERF1 shRNA-786 retrovirus or vector control. To facilitate tumour formation from T47D cells, which are oestrogen-dependent, we pre-implanted each mouse subcutaneously with a slow-release oestrogen pellet. Two days after pellet implantation, T47D/NHERF-786 or T47D/Babe cells were mixed with Matrigel and injected into the mouse mammary pads. Tumours were visibly established in mice 42 days after injection, when all mice were sacrificed to compare tumour size. As shown in Figure 4a and 4b, tumours formed from T47D/NHERF-786 (72.4 ± 13.9 mg, *n *= 10) were significantly larger than those from T47D/Babe cells (37.7 ± 8.8 mg, *n *= 8) (*P *= 0.043), suggesting that lowered NHERF1 expression promotes tumour growth *in vivo*. None of the tumours was found to metastasise to lung or liver.

### Effect of NHERF1 on cell cycle progression

**Figure 5:**
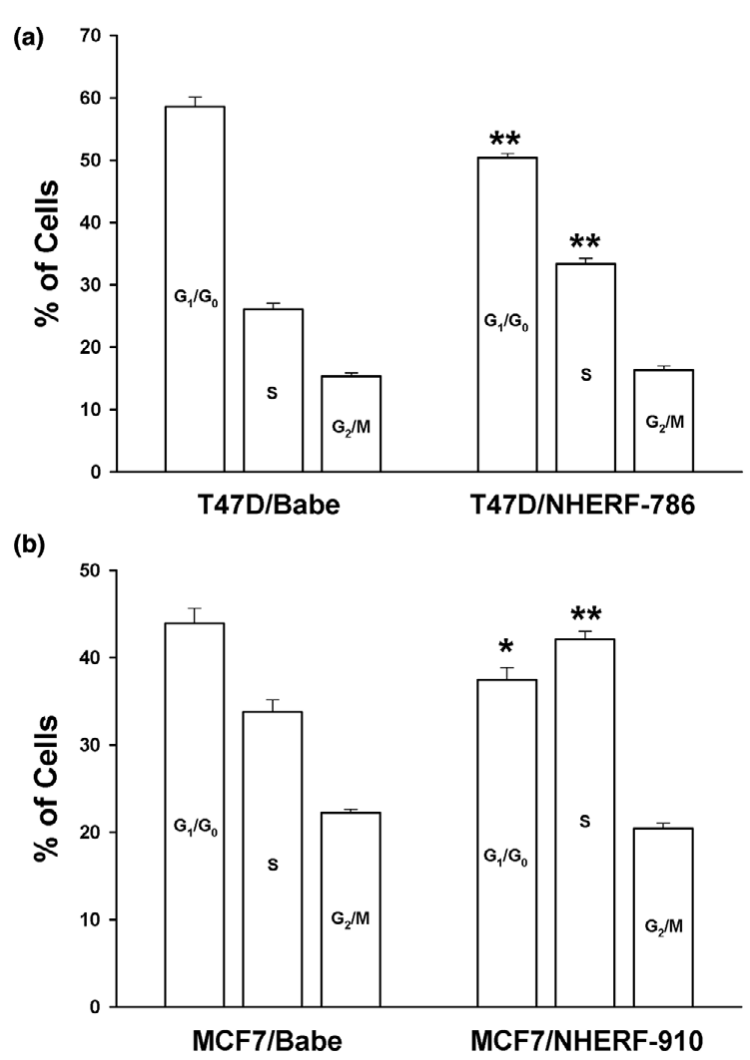
Effect of Na^+^/H^+ ^exchanger regulatory factor 1 (NHERF1) knockdown on cell cycle distribution. **(a) **The cell cycle distribution of asynchronous T47D cells (NHERF1 knockdown and Babe control) was determined by fluorescence-activated cell sorting (FACS) analyses. Cells were cultured in media supplemented in 10% foetal bovine serum before being trypsinised, fixed in 75% ethanol, and stained with propidium iodide. **(b) **Cell cycle distribution of MCF7/NHERF-910 and MCF7/Babe cells was assessed by FACS. Three independent experiments were conducted. Data are presented as means ± standard error. *P < 0.05, **P < 0.01, as compared with Babe cells.

To examine whether the growth-inhibitory activity of NHERF1 can be attributed to its effect on cell cycle progression, we first compared the cell cycle distribution of asynchronised NHERF1-negative and -positive T47D and MCF7 cells. As shown in Figure 5a, T47D/NHERF-786 cells had a lower G_1_/G_0 _and a higher S-phase population (G_1_/G_0 _50.4% ± 0.69%, S 33.3% ± 0.95%) than Babe control cells (G_1_/G_0 _58.6% ± 1.49%, S 26.0% ± 1.03%). No significant difference was observed in G_2_/M-phase populations between the two groups (16.3% ± 0.64% versus 15.4% ± 0.47%). Similar results were shown for MCF7 cells with NHERF1 knockdown (G_1_/G_0 _37.2% ± 1.69%, S 42.1% ± 0.98%, and G_2_/M 20.4% ± 0.60% for NHERF-910 versus G_1_/G_0 _43.9% ± 1.75%, S 33.8% ± 1.39%, and G_2_/M 22.3% ± 0.39% for Babe; Figure 5b), which suggested that NHERF1 affects cell cycle progression, probably by stalling cells at G_1 _phase.

**Figure 6:**
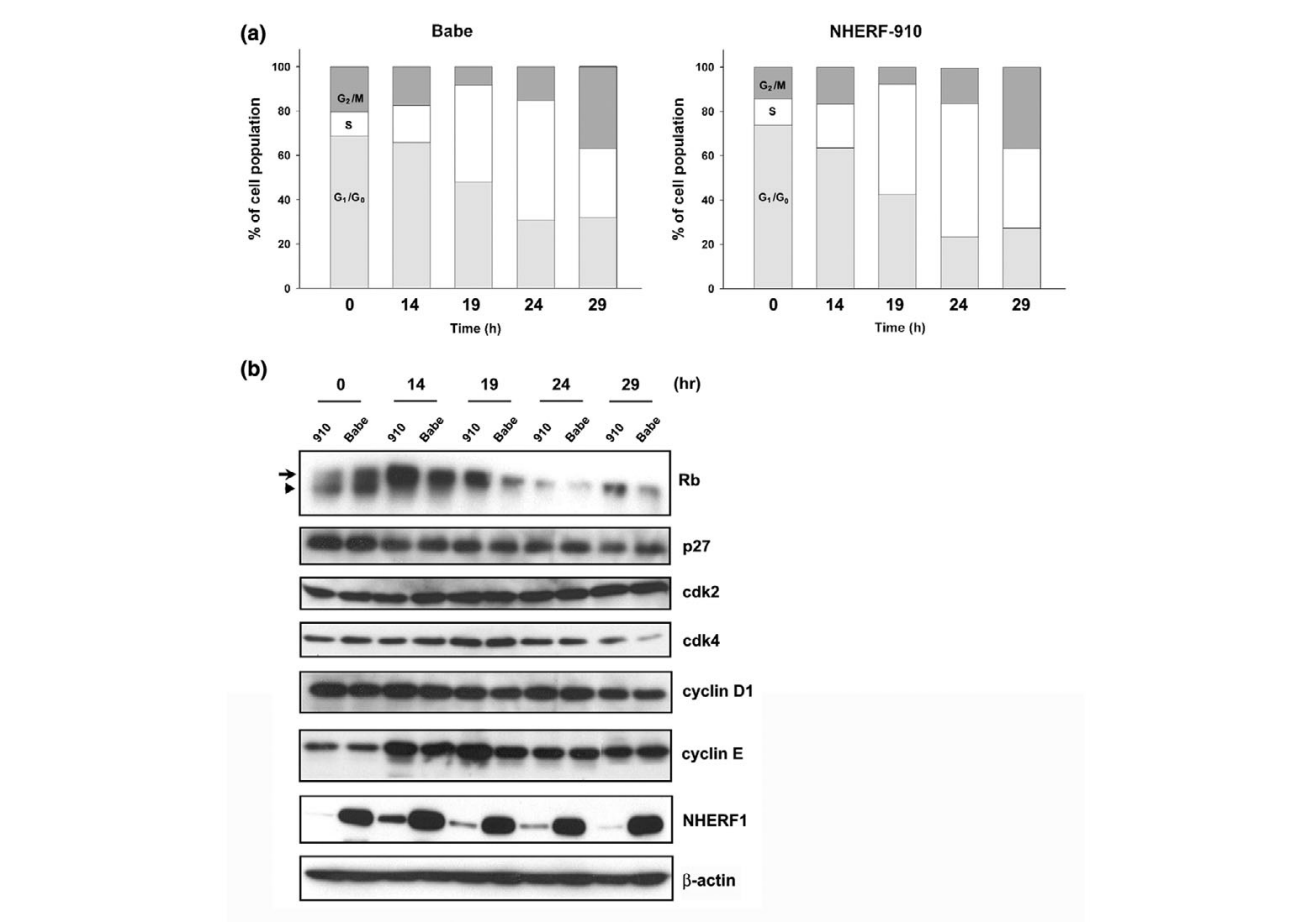
Stimulation of G_1_-to-S progression by Na^+^/H^+ ^exchanger regulatory factor 1 (NHERF1) knockdown. **(a) **MCF7/NHERF-910 and MCF7/Babe cells were cultured in serum-free media for 1 day to accumulate cells at G_1_/G_0 _phase. Cells were then re-fed with medium supplemented with 10% foetal bovine serum for 0, 14, 19, 24, and 29 hours, when cells were trypsinised for fluorescence-activated cell sorting analyses of cell cycle progression. **(b) **Cells harvested at various time points were lysed for immunoblotting analyses of Rb, p27, cdk2, cdk4, cyclin D1, and cyclin E expression. Hyper-phosphorylated and hypo-phosphorylated forms of Rb are indicated by arrow and arrowhead, respectively. Membranes were also probed with NHERF1 for knockdown verification and with β-actin for loading control. The result presented was representative of two independent experiments.

To analyse this possibility in more detail, we serum-starved MCF7 cells to arrest the cell cycle at the G_1_/G_0 _phase. One day of starvation led to accumulation of G_1 _phase from approximately 40% with normal serum condition to more than 70% with serum starvation. The cells were then re-fed with medium containing 10% serum and were allowed to grow for 0 to 29 hours before being harvested for FACS analyses (Figure 6a). At 14 hours after serum feeding, G_1_/G_0 _phase cells began advancing to the S phase. Between 14 and 19 hours, a remarkable increase occurred in the S-phase population. Comparing MCF7/Babe and MCF7/NHERF-910 cells, we found that NHERF-910 cells proceeded to S phase significantly faster than their corresponding control. At the 19-hour point, 43.2% and 50.0% of the Babe and NHERF-910 cells, respectively, were in S phase. This trend lasted through 24 hours after serum feeding. At 24 hours, S phase comprised 53.9% and 60.3% of the Babe and NHERF-910 cells, respectively (Figure 6a). In contrast, more cells remained at G_1 _phase in the Babe (47.8% and 30.8%) than in the NHERF-910 group (42.4% and 23.2%) at the 19- and 24-hour time points, respectively. These results indicated that NHERF1 prohibited G_1_-to-S cell cycle transition, reflecting the growth-inhibitory activity of NHERF1.

Phosphorylation of Rb protein is one of the most critical processes in enforcing the G_1_-to-S transition [34]. To further verify whether NHERF1 plays an inhibitory role in G_1_-to-S cell cycle progression, we compared the Rb protein expression in MCF7/Babe and MCF7/NHERF-910 cells. As shown in Figure 6b, the Rb protein in growth-arrested cells (at 0 hours) was mainly the hypo-phosphorylated form. Corresponding to the beginning of S-phase entry at 14 hours after serum feeding, we detected a high level of hyper-phosphorylated Rb, which gradually receded during the observation period. In consonance with an accelerated G_1_-to-S transition as a result of NHERF1 loss, MCF7/NHERF-910 cells contained a significantly higher level of hyper-phosphorylated Rb than did the Babe control, most prominently at 14 hours and 19 hours after serum feeding. To explore the mechanism responsible for the difference in Rb phosphorylation and G_1_-to-S transition, we compared the two groups of cells in their expression of some of the most prominent cell cycle regulatory proteins (Figure 6b). No difference was shown in the level of p27, cdk2, cdk4, or cyclin D1 between the MCF7/NHERF-910 and MCF7/Babe cells. However, we found that the cyclin E level in MCF7/NHERF-910 cells was significantly higher than that in MCF7/Babe at 14 hours and 19 hours after serum stimulation (Figure 6b). A similar result was obtained from another independent experiment. Taken together, these results indicated that NHERF1 knockdown leads to accelerated G_1_-to-S transition that may involve increased cyclin E content and elevated Rb phosphorylation status.

## Discussion

In the present study, we examined phenotypic changes in response to knockdown of endogenous NHERF1 expression by RNA interference. We found that the knockdown of NHERF1 in human breast cancer cells led to enhanced growth in either an anchorage-dependent or -independent manner. Our study was conducted on a tissue type most relevant to NHERF1 tumour suppressor activity. Results were verified in two breast cancer cell lines and by using two different shRNA targeting sequences. Coupled with our genetic evidence reported earlier, the current functional analyses substantiate NHERF1 as a tumour suppressor gene in mammary gland.

Phosphorylation of NHERF1 was shown to oscillate during cell cycle progression [35]. However, it was not clear whether NHERF1 plays a role in cell cycle regulation or how phospho-modification on NHERF1 affects cell cycle transition. The current study provides the first direct evidence indicating that the normal NHERF1 function may involve deceleration of the G_1_-to-S progression. The accelerated G_1_-to-S progression as a result of NHERF1 knockdown is accompanied by elevated Rb phosphorylation and cyclin E expression (Figure 6). Phosphorylation of Rb is believed to be triggered initially by cyclin D-dependent kinase and then accelerated by cyclin E-cdk2 complex [36,37]. An increase in cyclin E level as a result of NHERF1 loss may speed up the process of Rb phosphorylation and subsequent E2F-mediated gene transcription for S-phase entry. It is not clear at present how decreased NHERF1 expression enhances the cyclin E level. Given the contributing role of cyclin E in mammary gland hyperplasia and tumourigenesis [38,39], it is conceivable that the deregulation of cyclin E as a result of NHERF1 loss contributes to the breast cancer initiation or progression.

Human *NHERF1 *is thought to be an oestrogen-inducible gene; *NHERF1 *mRNA and protein were found to be inducible by oestrogen treatment, a response that is blocked by anti-oestrogen [24]. A few half-sites of oestrogen response element (ERE) at the 5'-regulatory sequences of the human *NHERF1 *gene were found to be responsible for its oestrogen-inducible expression [25]. In light of the key role of oestrogen in mammary gland development and mitogenic responses of many ER-α-positive breast cancer cells to oestrogen, it seems paradoxical that *NHERF1 *would act as a tumour suppressor gene in breast [22]. Our present study did not directly address the relation of NHERF1 to oestrogen. However, when we compared the NHERF1 shRNA and Babe cells (both T47D and MCF7), we found that NHERF1 expression status had no significant effect on oestrogenic responses measured by DNA synthesis and activation of ERE-driven reporter (our unpublished data), suggesting that NHERF1 is at least not an immediate mediator of classic oestrogen responses. Whether NHERF1 precipitates certain oestrogenic effects other than the canonical mitogenic responses remains to be determined. It should be pointed out that NHERF1 expression in breast cancer cells is not necessarily correlated with ER-α status. In agreement with observations of primary breast carcinoma [32], our panel of breast cancer cell lines revealed an inconsistent relationship between NHERF1 and ER-α positivity (Figure 1), suggesting that regulation of NHERF1 expression exists at levels other than oestrogen stimulation. Speculatively, alterations of these factors in mammary gland may cause an imbalance of NHERF1 level that could lead to neoplasia. Interestingly, the mouse *NHERF1 *gene does not contain the ERE sites found in human *NHERF1*, and as a result, mouse NHERF1 expression did not respond to oestrogen [40], suggesting a difference in transcriptional regulation among species to control NHERF1 expression.

The study presented here recapitulated the putative tumour suppressor activity of NHERF1 in a cell culture model. A true test of the NHERF1 effect on mammary tumourigenesis, however, would be to analyse mammary gland development and susceptibility of mammary carcinogenesis in *NHERF1 *knockout mice [41]. Recently, we found that NHERF1^-/- ^mice displayed elevated ductal side branching and extensive mammary gland hyperplasia (our unpublished data). This observation is consistent with the data of this study, which indicate that NHERF1 suppresses cell growth at the mammary site. Whether the disturbance of mammary gland development as a result of *NHERF1 *gene loss is sufficient to increase breast cancer incidence needs to be investigated.

Although our study addressed the biologic effect of NHERF1 on the proliferation of breast cancer cells, it remains unclear which NHERF1-associated pathway, among all NHERF1-interacting partners, is responsible for the NHERF1 tumour suppressor function. We reported earlier that NHERF1 interacted with SYK and merlin [1]. The tumourigenic mutations of NHERF1 partially or completely disrupt the binding of SYK or merlin, both of which are tumour suppressors [19,20,42,43], suggesting that NHERF1 converges in a pathway mediated by the two tumour suppressors. Recently, NHERF1 was reported to interact with PDGFR and PTEN (phosphatase and tensin homologue [mutated in multiple advanced cancers 1]), forming a ternary complex [44]. NHERF1 was hypothesised to assist in recruitment of PTEN to attenuate the PI3K (phosphoinositide-3 kinase) activity initiated by PDGF. Although the hypothesis contrasts with the cooperative effect of NHERF1 on PDGF signaling as suggested by some earlier studies [9,21], this mechanism is consistent with the tumour suppressor activity presented in this study. Whether the negative regulation of growth factor signaling by NHERF1 is responsible for the NHERF1 tumour suppressor function in mammary gland remains to be determined.

## Conclusion

By functional analyses, we show for the first time that NHERF1 possesses growth suppressor activity. We further show that the G_1_-to-S cell cycle progression is accelerated by NHERF1 knockdown, a phenotype that is accompanied by increased levels of cyclin E and phosphorylated Rb. Based on our published genetic evidence implicating NHERF1 as a tumour suppressor gene [1], we propose that one of the functional activities responsible for its tumour suppressor role is proliferative suppression of mammary epithelial cells. Together, our findings suggest that NHERF1 constitutes a proliferation control pathway in breast cells, justifying further studies of a novel pathway that may represent a potential opportunity for therapeutic intervention.

## Abbreviations

ER = oestrogen receptor; ERE = oestrogen response element; ERM = ezrin-radixin-moesin; FACS = fluorescence-activated cell sorting; FBS = foetal bovine serum; LOH = loss of heterozygosity; MTT = 3-(4,5-dimethylthiazol-2-yl)-2,5-diphenyltetrazolium bromide; NF2 = neurofibromatosis-2; NHE3 = Na^+^/H^+ ^exchanger isoform 3; NHERF1 = Na^+^/H^+ ^exchanger regulatory factor 1; PBS = phosphate-buffered saline; PDGFR = platelet-derived growth factor receptor; PDZ = PSD-95/Dlg/ZO1; PTEN = phosphatase and tensin homologue (mutated in multiple advanced cancers 1); shRNA = short hairpin RNA; SYK = spleen tyrosine kinase.

## Competing interests

The authors declare that they have no competing interests.

## Authors' contributions

YP participated in the experimental design and interpretation of results, carried out experimental procedures, and drafted the manuscript. LW participated in the experimental design. JLD participated in the experimental design and interpretation of results and assisted in writing and editing the manuscript. All authors read and approved the final manuscript.
